# Perish or Publish Dilemma: Challenges to Responsible Authorship

**DOI:** 10.3390/medicina56030123

**Published:** 2020-03-12

**Authors:** Vygintas Aliukonis, Margarita Poškutė, Eugenijus Gefenas

**Affiliations:** Centre for Health Ethics, Law and History, Institute of Health Sciences, Faculty of Medicine, Vilnius University, 03101 Vilnius, Lithuania; vygintas.aliukonis@mf.vu.lt (V.A.); margarita.poskute@mf.vu.lt (M.P.)

**Keywords:** authorship, authorship misuse, honorary authorship, ghost authorship, publication ethics, research integrity

## Abstract

Controversies related to the concept and practice of responsible authorship and its misuse have been among the most prominent issues discussed in the recent literature on research integrity. Therefore, this paper aims to address the factors that lead to two major types of unethical authorship, namely, honorary and ghost authorship. It also highlights negative consequences of authorship misuse and provides a critical analysis of different authorship guidelines, including a recent debate on the amendments of the International Committee of Medical Journal Editors (ICMJE) authorship definition. Empirical studies revealed that honorary authorship was the most prevalent deviation from the responsible authorship standards. Three different modalities of honorary authorship were distinguished: *gift authorship*, *guest authorship*, and *coercive authorship*. Prevalence of authorship misuse worldwide and in Europe was alarmingly high, covering approximately one third of all scientific publications. No significant differences were reported in authorship misuse between different health research disciplines. The studies conducted in North America highlighted the most effective means to cope with unethical authorship. These were training in publishing ethics, clear authorship policies developed by medical schools, and explicit compliance with the authorship criteria required by the medical journals. In conclusion, more empirical research is needed to raise awareness of the high prevalence of authorship misuse among scientists. Research integrity training courses, including publication ethics and authorship issues should be integrated into the curricula for students and young researchers in medical schools. Last but not least, further discussion on responsible authorship criteria and practice should be initiated.

## 1. Introduction

Modern health care research must be navigated within the complex framework of normative guidelines. This framework covers two major fields of rather different, however, interconnected ethical issues. On the one hand, researchers must protect the rights and welfare of research participants. This is the core of what has been called research ethics since the emergence of the Nuremberg Code in 1948 and the adoption of the first version of the Declaration of Helsinki in 1964. On the other hand, another set of normative issues has become very prominent since the beginning of the 21th century. These are research integrity concerns focusing on research misconduct cases, such as fabrication or falsification of research data, and plagiarism as well as the so-called questionable research practices, such as mentorship, conflicts of interest and responsible authorship to mention but a few.

In this paper, we have concentrated on the controversies related to the concept and practice of responsible authorship and its misuse, which has recently been among the most prominent issues discussed in the literature on research integrity. Education rather than sanctions has been seen as a more important means to change the culture and entrenched habits in the field of scientific writing. Therefore, this issue is located closer to the ethical rather than legal end of the whole spectrum of research integrity concerns. In addition, a consensus on the definition of responsible authorship has not yet been reached. That is why the authorship issues have been categorized as questionable research practices to distinguish them from research misconduct cases, such as legally penalized practices of fabrication and falsification of study results, like the widely discussed data fabrication case of Dutch social psychologist D. Stapel [1].

To address the factors that lead to unethical authorship, we conducted a literature review. We first addressed the importance of the authorship issue and highlighted the emerging controversy surrounding the International Committee of Medical Journal Editors (ICMJE) authorship definition. We then focused on two major types of unethical authorship practices, namely, honorary and ghost authorship, and addressed the motivation behind these practices and their negative consequences. We continued with a critical analysis of different authorship guidelines and emerging discrepancies between them. Finally, yet importantly, we have presented recommendations on how to increase compliance with the criteria of responsible authorship and invited the reconsideration of ICMJE authorship recommendations.

## 2. Why Does the Issue of Authorship Deserve Special Attention?

Authorship has for a long time been the principal means of recognizing academic achievements in the world of modern science. The list of publications is one of the main criteria influencing academic carriers and distributing research grants. The well-known “publish or perish” dilemma highlights the main incentive fueling the need to publish as much as possible, preferably in high impact journals. Those who fail in this competition may also miss career opportunities, research funding or other rewards [2].

The competitive environment can also explain why non-compliance with the requirements of responsible authorship is a global phenomenon affecting both developed countries as well as countries transitioning to transparent and democratic governance. However, in the latter countries a scientific community is still in the initial stage of building a culture of responsible conduct of research and transparent interaction between senior and junior researchers. Therefore, in these countries it can be more difficult to achieve a compliance with the criteria of responsible authorship, as described in Section 6 of this paper.

There are several general reasons why compliance with responsible authorship guidelines is a complicated process. One important challenge to follow responsible authorship guidelines and practices comes from a hierarchical relationship within academia where senior members of staff exert power on junior members [3]. In such setting, a tacit requirement to attribute the authorship to the chief of the division or even the department can be imposed on junior collaborators. In another case and in contrast to the previous example, true authors may not even appear among the authors to “hide” their conflicts of interest and/or industry ties.

Another set of challenges is related to the definition of authorship as such. At first glance, the concept of authorship seems to be clear and an uncomplicated one. For example, many influential medical journals follow Recommendations for the Conduct, Reporting, Editing, and Publication of Scholarly work in medical journals (commonly referred to as the Vancouver Convention) issued by the ICMJE. Recommendations provide for a seemingly simple definition of authorship based on four necessary conditions, which are:

1. Substantial contributions to the conception or design of the work; or the acquisition, analysis, or interpretation of data for the work;
2. Drafting the work or revising it critically for important intellectual content;
3. Final approval of the version to be published;
4. Agreement to be accountable for all aspects of the work in ensuring that questions related to the accuracy or integrity of any part of the work are appropriately investigated and resolved [4].

This simplicity is, however, rather deceptive. For example, the ICMJE guidelines have been challenged as being too restrictive because of the second condition, which is particularly problematic to comply with in the context of multidisciplinary research that requires the participation of different experts not all of whom should be directly involved in the process of drafting or revising the manuscript. Recent debate in the academic literature has flagged this controversy and has shown the need for conceptual amendments reflecting the realistic contribution of those participating in the processes of scientific writing [5,6,7]. However, before addressing this issue in more detail, we have made an overview of more general issues related to authorship misuse.

## 3. Types of Unethical Authorship

There are two dominant types of authorship misuse taking completely different manifestations. The first one deals with “honorary” or “undeserved” authorship [8]—terms that already appeared in Stewart and Federer’s paper in 1987. The second type of unjustified authorship is the so-called “ghost” authorship referring to situations when somebody tries to hide his or her involvement in the scientific work.

Honorary authorship is the most prevalent deviation from the responsible authorship standards. In this case, a person who has not contributed at all to or was just marginally involved in the scientific work appears among the listed authors. Luiten et al. [9] referred to three subtypes or modalities of honorary authorship reflecting different motives and incentives behind it. For example, in the case of a “*gift authorship*” someone is listed as a co-author “out of respect for or gratitude to an individual” [9] (p. 697). It emerges because the actual author hopes that the honorary author will return him or her the favor by quoting or including them in the list of co-authors in other publications. The second modality of honorary authorship is “*guest authorship*”. In this case a well-known researcher is invited to become a co-author hoping to increase the apparent quality of a paper or to just to hide “a paper’s industry ties by including an academic author” [9] (p. 697). Finally, “*coercive authorship*” occurs when a senior researcher forces a junior one to include a gift or guest author [9] (p. 697). Coercive authorship looks like the most severe deviation from the responsible authorship practice because it does not only include undeserved attribution of authorship but also involves undue influence on the junior part of the interaction. It very often stems from a tradition and hierarchies still existing in many academic settings. It should be noted that three modalities of honorary authorship—gift, guest, and coercive authorship—can overlap.

In contrast to different modalities of honorary authorship, the ghost author is usually defined as a person who contributed substantially to an article but is not mentioned as an author or co-author of the resulting publication [5,10]. Ghost authorship may also appear in different contexts: from pharmaceutical companies paying professional writers for their articles to students ordering someone to write a masters thesis or a case of a politician using ghost-writers for their speeches.

## 4. How Widely Is Authorship Misuse Spread?

To better understand the prevalence and origins of authorship misuse, it is useful to look at this phenomenon from different perspectives. Therefore, the correlation of authorship misuse with multiple authorship, prevalence across different geographical areas, medical fields, and journals depending on their impact factor are the issues to be briefly explored.

Up until 1955 it was much more usual for one person to be an author of a scientific paper [11], which did not leave much room for honorary authorship. However, between 1980 and 2000 the average number of authors in four leading medical journals increased by 53% [12]. It seems there is no causal relationship between multi-authorship and ethically dubious authorship, however, a higher number of authors provides more room to manipulate the authorship criteria. In addition, multi-authorship requires the determining of the sequence of co-authors in the by-line reflecting their scientific contribution [13,14,15], another complex issue which deserves separate consideration.

An interesting tendency was revealed by empirical studies on authorship misuse in different geographical areas and countries. Eisenberg et al. [16] found a significantly higher rate of honorary authorship in radiology journals reported by the first authors of scientific papers in Asia (38.9%) or Europe (34.3%) as compared to North America (19.1%), the intercontinental average being 27.7%. Furthermore, according to the same study, 40.0% of the section or department heads in Europe and 47.2% in Asia were automatically listed as co-authors in the publications, as compared to 6.6% in North America [16]. Similar tendencies were revealed by a meta-analysis of 14 studies on authorship issues performed by Marušić et al. revealing an average of 29% researchers reporting their own or others’ experience with authorship misuse worldwide [17]. This study also showed uneven trends in geographical distribution of authorship misuse: 23% of authors reported authorship misuse in USA/UK or international journal settings, while non-compliance with the authorship requirement in France, South Africa, India and Bangladesh was reported to be as high as 55% [17]. On the other hand, studies conducted in the Nordic countries revealed a lower level of authorship misuse as compared to the European average. For example, a survey of all doctoral students attending basic PhD courses at medical faculties in Stockholm and Oslo during the fall of 2014 showed that about 23% of the respondents may have experienced unethical pressure concerning the order of authors [18]. It is worthwhile to note that the comparison of the results with previous studies also showed that unethical inclusion and ordering of authors was an increasing problem in Nordic countries [18,19].

It appeared that honorary authorship was much more common in low- and middle-income countries where local researchers tended to co-author papers with some renowned authors from high ranked Western research institutions [20]. Similarly, in countries with predominantly non-indexed journals, such as Iran, honorary authors were present in 89% of papers in medical journals [21]. On the other hand, a survey of 630 corresponding authors from high-impact journals such as *the Annals of Internal Medicine*, *JAMA*, *The Lancet*, *Nature Medicine*, *The New England Journal of Medicine* and *PLoS Medicine* revealed the average prevalence of both honorary and ghost authorship of 21% [22]. Although this is significantly less than compared to the findings from the previous example, the spread of authorship misuse in high impact journals is still rather high.

No major differences seem to exist in the frequency of authorship misuse across different health care fields. Authorship misuse is rather equally spread across the medical journals within radiology (from 27.7% to 50.3%) [16], general surgery (from 15.0% to 44.0%) [9], dermatology (14.3% to 41.4%) [23], spine-dedicated journals (49.1%) [24], and nursing (42%) [25].

As compared to honorary authorship, the number of studies focusing on the prevalence of exclusively ghost authorship was much lower. The prevalence itself, however, was shown to vary considerably. For example, Marušić et al. found [17] that the prevalence of ghost authorship ranged from 2% to 75% as reported in different studies. This type of authorship misuse seemed to be a rather neglected one, as it was not considered by the editors of the medical journals as a serious problem and there were rather few academic medical centers in the countries with relatively developed authorship policies, such as USA, explicitly banning ghostwriting [17].

## 5. Negative Consequences of Authorship Misuse

Sustaining trust between the general public and scientists is a necessary condition to facilitate public understanding of science as well as informed public debate on new technologies and scientific developments. Laypeople rely on scientific experts when developing their views, therefore a violation of research integrity, including unethical authorship, damages trust between the general public and scientific experts. Trustful relationship between the researchers themselves is also a fundamental condition for successful development of science, since contemporary multidisciplinary research requires knowledge generated by many experts coming from different fields [26]. To sum up, honorary authorship harms trust in science because honorary authors cannot take full responsibility for the study results and therefore “undermine trust and confidence in the quality of research itself and the reputation of the research community more generally” [27] (p. 6).

Besides damaging trust between the society and the scientific community, honorary authorship also harms the true authors by diluting their contribution and giving undeserved competitive advantage to unethical researchers by facilitating their promotion and access to research grants [28,29]. Authorship is not a sole inscription of one’s surname on the author line. As quote-based bibliometrics gives “citation credit” to all of the listed authors, no matter how many there are, scientific weight of honorary authors grows in parallel with their citation volume and creates unfair competitive advantage on a broader scale [30] (p. 237).

There are also some specific organizational features of authorship misuse leading to structural effects that make it even more difficult to prevent this practice. One such feature is “transformation” of academic capital, such as administrative power or seniority, into the honorary authorship [31]. The problem is that transforming the seniors into honorary authors forms a vicious circle, which can be difficult to interrupt because it may also benefit the actual author. The growing number of publications affiliated with the actual author’s institution increases its prestige and visibility and at the same the chances of the institution and its representatives to attract funding for further research. It should be noted that incentives for honorary authorship are strengthened by the pressure to search for funds from external resource providers. This pressure, which is a manifestation of what Slaughter et al. [32] described as “academic capitalism”, is faced nowadays by most of the academic institutions. 

Kovacs [31] also revealed another tendency of having a systematic effect. Honorary authorship can be shared not only between individuals but also between different groups of academics establishing so-called “publication cartels”. These cartels emerge when different groups of authors working on similar topics add each other’s names to the papers to be published. The interaction between such groups of authors does not necessarily imply that an undeserved author would be included in the byline [31], however, the probability that they would be involved in the gifted authorship modality is higher. Both mentioned tendencies —the exchange between academic capital and honorary authorship, as well as forming the publication cartels—remarkably strengthen the negative impact of authorship misuse.

Although the nature of ghost authorship differs from that of honorary authorship, it leads to similar negative consequences. In many cases, the issue of ghost authorship overlaps with the conflict of interest and breaks the confidence in science. The World Association of Medical Editors (WAME) refers to the main threat of ghost authorship as “to persuade readers in favor of a special interest” [33]. Moreover, hiding a scientist’s name may also hide research misconduct, such as data fabrication or falsification [34]. As a result, study results published in peer-reviewed journals may be distorted by the bias of different interest groups thus leading to loss of credibility. Even more importantly, it can have a detrimental impact on clinical decisions and the well-being of patients.

## 6. Training and Other Means to Prevent Authorship Misuse

Authorship misuse can happen not only due to a conscious decision influenced by power relations. It may also occur because of ignorance or insufficient awareness of the authorship rules [25,35]. In other words, some cases of honorary authorship may not be perceived as authorship misuse because researchers simply are not informed about authorship requirements. For example, a survey of postgraduate medical trainees conducted by Rajasekaran et al. [36] in Canada revealed that 38.1% of the respondents positively answered a generic question about inclusion of an honorary author (an individual who has not made substantial contributions as an author) in a previous poster/podium presentation or manuscript. On the other hand, a much higher percentage (57%) of the same respondents acknowledged the inclusion of honorary authors when they were explicitly asked if the co-authors satisfied all four ICMJE authorship criteria. The difference between the rate of what was initially perceived by the respondents as honorary authorship and the ”ICMJE-defined honorary authorship” was explained by the finding that more than 90% of the respondents were unaware of the ICMJE authorship criteria. At the same time, they thought that medical trainees and faculty should be instructed on authorship guidelines. This highlighted the importance of training to prevent the authorship misuse. A study by Eisenberg et al. [16] referred earlier also supported this conclusion. It revealed that authorship violations were less likely to happen in North America due to training in publishing ethics: only 13% respondents from the institutions that provided lectures or courses on publication ethics reported about honorary authorship as compared with 35% of respondents whose institutions did not offer such courses. This is why medical schools and universities should play a much more active role in introducing training modules on research integrity into teaching programs.

The absence of explicit references to authorship guidelines in the editorial policies of biomedical journals may also perpetuate a high prevalence of honorary authorship. This point can be particularly relevant in the transition countries as was demonstrated by Broga et al. [37]. The study using the data from the English language biomedical journals identified in Medline showed that only 40% of the journals in the Eastern and Central European countries of the European Union, such as Czech Republic, Hungary, Romania, Bulgaria, Slovenia, Slovakia, Poland, Lithuania, Latvia and Estonia had adopted authorship policy. Although the situation might have improved since 2011 when the study was performed, some general trends conducive to the high prevalence of authorship misuse might still be valid. For example, an overview of 14 indexed medical journals with an on-line access that were included in the Lithuanian Library of Medicine database [38] revealed a rather diverse picture regarding the inclusion of the explicit authorship criteria into their guidelines for authors. Only three journals provided an explicit list of the authorship criteria together with a reference to the international authorship guidelines, such as ICMJE Recommendations. Six journals provided only very general information on the authorship criteria (e.g., mentioning the requirement of substantial contribution) or referred to other authorship guidelines without providing any information about the authorship criteria. Finally, 5 out of 14 journals had no reference to the authorship guidelines or criteria at all.

In addition to the training needs and explicit authorship policies to be required by the medical journals, the Canadian study [36] revealed that an overwhelming majority (92%) of the postgraduate medical trainees believed that a support system for the authorship disputes should be implemented at the institution. The need to establish a system that helps resolve authorship disputes was also emphasized by other studies [39]. Such a system might encourage open discussions about the roles of different team members and corresponding authorship issues even before the commencement of a research project. It may be helpful not only in clarifying basic rules and concepts but also in creating a transparent environment at the institution. Finally, a system to resolve authorship disputes should be a part of the broader authorship policy adopted by the institution. The lack of information about authorship policy is relevant even for countries with relatively low rates of authorship misuse. As stated by Lacasse et al. [40], even in the U.S. only a small part out of 50 influential academic medical centers published authorship criteria that reflected ICMJE recommendations. It was noted that a good practice of building institutional authorship policy would involve all academic staff at the universities, including postdoctoral scholars, graduate and undergraduate students, into the process of developing, distributing and regularly reviewing their policies on authorship [5].

Although ghost authorship does not usually occur due to the ignorance of the rules, awareness of authorship guidelines and relevant training is also important in this context. In medical articles, most of the ghost authors are employed or contracted by the pharmaceutical companies and as such profit directly [41]. However, the influence of pharmaceutical companies would be less effective if the biomedical community was better informed about the ethical authorship practices.

A dialogue and sharing of information on the responsible authorship issues promoted during different international fora, such as the International Congresses on Peer Review and Scientific Publication [42] or the World Conferences on Research Integrity [43] should be mentioned in this context. Among other topics, these meetings are an important world-wide means to fill in informational gaps in this field.

## 7. Critical Analysis of the Leading Authorship Guidelines

As noted earlier, one of the most influential authorship guidelines have been adopted by the ICMJE. The ICMJE Recommendations have been followed by a number of high impact medical journals, including *the Lancet*, *the British medical journal*, *the JAMA Network journals* [44], and even the social sciences journals [45]. However, it appears that despite being very influential and widely accepted by the biomedical scientific community, the ICMJE authorship criteria have sparked a debate about their applicability in the real world of academic writing.

Most of the academic debate has been targeting the requirement to be involved in drafting or revising a manuscript critically for important intellectual content, which is the second of the necessary authorship conditions of the ICMJE recommendations. It is intuitively appealing to follow this condition because a generic idea of authorship as defined in the Cambridge dictionary is “the state or fact of being the person who wrote a particular book, article, play, etc.” [46]. However, although an involvement in the writing process seems to be a core feature of being an author, it can also be seen as a rather limiting one in the context of scientific publishing.

For example, members of a large multidisciplinary research team can have very different roles in the project. Involvement in drafting or revising a manuscript should be seen not as a necessary condition but just as one function comparable to other roles, such as acquisition and analysis of data or computing skills. It seems therefore that a requirement to involve every single member of the team into the drafting or revising of a manuscript is too ambitious. If applied literally, it can lead to the exclusion of some members of the research team (e.g., computer programmer) from the authors list. In practice, however, these co-workers are listed as authors due to their significant impact, which formally violates the ICMJE authorship criteria. Unfortunately, such a situation weakens the authority of the important guidelines. It also paves the way to a scenario of honorary inclusion of underserved authors, such as senior researchers or heads of the departments who were just marginally involved in the research project, and the exclusion from the authors list of the computer programmers who constructed the key algorithms for the project [7].

## 8. Alternative Definitions of Authorship

A prominent alternative definition of authorship was recently suggested by McNutt et al. [5]. This alternative significantly broadens the eligible activities of authors because it merges the first and the second ICMJE requirements. In this way the involvement in drafting or revising a manuscript becomes just one among a few other possible authorship roles. According to this alternative definition: “Each author is expected to have made substantial contributions to the conception or design of the work; or the acquisition, analysis, or interpretation of data; or the creation of new software used in the work; or have drafted the work or substantively revised it; AND to have approved the submitted version (and any substantially modified version that involves the author’s contribution to the study); AND to have agreed both to be personally accountable for the author’s own contributions and to ensure that questions related to the accuracy or integrity of any part of the work, even ones in which the author was not personally involved, are appropriately investigated, resolved, and the resolution documented in the literature [5].”

Although the difference between the ICMJE and the McNutt et al. criteria seems rather insignificant from a linguistic point of view, it brings an important practical change. It is clear that similarly to the ICMJE Recommendations, the McNutt definition retains the same fundamental requirements of being responsible for the content, accuracy and integrity of the whole work. On the other hand, McNutt’s definition expands legitimate roles of a potential author as it includes five different roles into its first requirement. This change makes authorship guidelines not writing-mandatory [6] and diversifies the roles of the authors offering more flexibility to compose a research team where all its members would afterwards be legitimate authors of the paper. It should be noted that although the attraction of research funding is a very important precondition to carry on research activities, it is not a sufficient ground for the attribution of authorship. The attraction of research funding is rather an item to be included in the acknowledgement section of the paper. To sum up, amending the authorship criteria can have significant practical and ethical implications. It allows to better specify the contribution of each author involved in the work. This can make the evaluation of the impact of each of the authors more nuanced and accurate as it reflects the actual work done. In this case there is no need to only formally comply with such authorship requirements as a direct involvement in the manuscript drafting or revising process, as is the case with the ICMJE guidelines. Therefore, adopting more realistic authorship criteria can lead to a reduction in honorary authorship. In addition to the ethical and research integrity dimension, an accurate attribution of authorship can also help to identify effective individual researchers and improve the process of composing the right mix of researchers needed to advance a particular research project in the context of modern science [6]. Many prominent publishers, such as *Cell Press*, *BMJ* or *Health & Medical Publishing Group* [47] as well as *Nature* journals [48] have already adopted the McNutt et al. recommendations [6].

## 9. Conclusions

There is a consensus in the academic literature that some measures to cope with the problem of authorship misuse should be taken as soon as possible. First, more empirical research is needed to raise awareness on the high prevalence of authorship misuse among scientists in many European countries. Empirical studies can also reveal the factors facilitating the wide spread of this phenomenon, thereby contributing to the development of a more efficient means to prevent it.

Second, training courses on research integrity including publication ethics and authorship issues should be integrated into the curriculum of medical students and young researchers. As have been pointed out, a large number of authors, even in highly ranked science and medical centers, were not only unaware of what was said in the most popular authorship guidelines, but were also unfamiliar with the existence of such guidelines.

Third, discussion on responsible authorship practices and guidelines should be initiated and the procedures for resolution of authorship disputes established at the institutional level.

Finally, as has been emphasized by comparing ICMJE and McNutt authorship criteria, the scientific community needs balanced and realistic authorship guidelines, which can also prevent authorship misuse and facilitate ethically sound and effective collaboration between scientists and their groups.
